# Genetic Contribution to Initial and Progressive Alcohol Intake Among Recombinant Inbred Strains of Mice

**DOI:** 10.3389/fgene.2018.00370

**Published:** 2018-09-25

**Authors:** Megan K. Mulligan, Wenyuan Zhao, Morgan Dickerson, Danny Arends, Pjotr Prins, Sonia A. Cavigelli, Elena Terenina, Pierre Mormede, Lu Lu, Byron C. Jones

**Affiliations:** ^1^Department of Genetics, Genomics, and Informatics, The University of Tennessee Health Science Center, Memphis, TN, United States; ^2^Albrecht Daniel Thaer-Institut für Agrar- und Gartenbauwissenschaften, Humboldt-Universität zu Berlin, Berlin, Germany; ^3^Biomedical Genetics, University Medical Center Utrecht, Utrecht, Netherlands; ^4^Department of BioBehavioral Health, The Pennsylvania State University, University Park, PA, United States; ^5^GenPhySE, INRA, ENVT, Université de Toulouse, Castanet-Tolosan, France

**Keywords:** BXD, DID, QTL, alcoholism, alcohol intake, genetic variation, B6, D2

## Abstract

We profiled individual differences in alcohol consumption upon initial exposure and during 5 weeks of voluntary alcohol intake in female mice from 39 BXD recombinant inbred strains and parents using the drinking in the dark (DID) method. In this paradigm, a single bottle of 20% (v/v) alcohol was presented as the sole liquid source for 2 or 4 h starting 3 h into the dark cycle. For 3 consecutive days mice had access to alcohol for 2 h followed by a 4th day of 4 h access and 3 intervening days where alcohol was not offered. We followed this regime for 5 weeks. For most strains, 2 or 4 h alcohol intake increased over the 5-week period, with some strains demonstrating greatly increased intake. There was considerable and heritable genetic variation in alcohol consumption upon initial early and sustained weekly exposure. Two different mapping algorithms were used to identify QTLs associated with alcohol intake and only QTLs detected by both methods were considered further. Multiple suggestive QTLs for alcohol intake on chromosomes (Chrs) 2, 6, and 12 were identified for the first 4 h exposure. Suggestive QTLs for sustained intake during later weeks were identified on Chrs 4 and 8. Thirty high priority candidate genes, including *Entpd2, Per3*, and *Fto* were nominated for early and sustained alcohol intake QTLs. In addition, a suggestive QTL on Chr 15 was detected for change in 2 h alcohol intake over the duration of the study and *Adcy8* was identified as a strong candidate gene. Bioinformatic analyses revealed that early and sustained alcohol intake is likely driven by genes and pathways involved in signaling, and/or immune and metabolic function, while a combination of epigenetic factors related to alcohol experience and genetic factors likely drives progressive alcohol intake.

## Introduction

According to Cloninger (1987) and Babor et al. (1992) there are multiple types of alcohol use disorders and likely different genetic contributions to each type. For each, though there is an initiating event, usually voluntary consumption and subsequent developments from avoidance to steady-state or ever increasing consumption and associated problems.

Genetic populations of animals have been used to model individual differences in the propensity to consume alcohol. One murine population that has contributed greatly to alcohol and addiction research is the BXD recombinant inbred family derived from C57BL/6J (B6) and DBA/2J (D2) inbred strains. Mice from these parental strains were crossbred to produce an F_1_ generation and then this generation was interbred for several generations to produce genetically segregating stocks. Next, families were selected for inbreeding to fix the alleles. The approach is described in more detail by Peirce et al. (2004). The result is a large number of inbred strains in which the alleles from B6 and D2 were recombined and redistributed. The BXD strains are segregating over five million variants that distinguish the parental B6 and D2 strains and all of the strains have been densely genotyped. Over 10 brain regions have been subjected to microarray analysis of gene expression and there is a freely available database^1^ consisting of more than 5,000 phenotypes contributed by many laboratories. This includes over 15 alcohol-related data sets in which the BXD family has been used to measure alcohol acceptance, consumption and preference (Phillips et al., 1994; Rodriguez et al., 1994; Gill et al., 1996; Fernandez et al., 1999); metabolism (Browman and Crabbe, 2000; Grisel et al., 2002; Philip et al., 2010); hypothermia, withdrawal, tolerance, and sensitivity (Belknap et al., 1993; Roberts et al., 1995; Crabbe et al., 1996; Phillips et al., 1996; Buck et al., 1997; Crabbe, 1998; Browman and Crabbe, 2000; Philip et al., 2010); locomotor response (Phillips et al., 1995; Browman and Crabbe, 2000; Philip et al., 2010); ethanol induced conditioned taste aversion (Risinger and Cunningham, 1998); and ethanol conditioned place preference (Cunningham, 1995). This family also contributed to the most detailed meta-analysis of genes that contribute to the predisposition for high alcohol consumption (Mulligan et al., 2006).

The research that we present here is a continuation of these studies in which we report on initial early intake and sustained alcohol consumption over a 5-week period in 39 BXD strains using the “drinking in the dark” (DID) protocol described by Rhodes et al. (2005). This protocol is used to elicit high alcohol consumption over a short time span and has been used to model binge-like alcohol consumption. There was considerable genetic variation among inbred strains of mice in alcohol intake using the DID paradigm (Rhodes et al., 2007; Crabbe et al., 2014). However, the DID procedure has typically been used to measure intake during a single week and has never been used to measure alcohol intake over multiple weeks in a recombinant inbred population. Here, we leverage the BXD family and accompanying legacy molecular, alcohol-trait, and other existing phenotypes to provide a systems genetics analysis of the factors driving alcohol consumption in the BXD family.

## Materials and Methods

### Animals

The subjects were female mice from 39 BXD recombinant inbred mouse strains and the two parental strains for the BXDs, B6 and D2 (within strain replicates ranged from 1 to 16, **Supplementary Table S1**). The animals were 60–80 days old at the start of the study. The mice were individually housed and fed a standard laboratory diet (Harlan Teklad 7912) with food and water available *ad libitum* except during exposure to alcohol (see below). The light cycle was 23:00 h lights on and 11:00 h lights off. This light cycle facilitated alcohol administration and measurement of alcohol intake. Mice were weighed weekly, and all procedures included here were approved by the UTHSC Institutional Animal Care and Use Committee.

### Drinking in the Dark

Alcohol consumption was evaluated using the DID method (Rhodes et al., 2005). The protocol calls for 4 consecutive days of testing. Each day, starting on a Tuesday 3 h after lights were turned off, the water bottles were removed from the cages and replaced with 15 ml centrifuge tubes filled with 20% (v/v) ethanol from 95% USP ethanol. On days 1–3 (Tuesday through Thursday) the length of exposure was 2 h and on the 4th day (Friday) the exposure was 4 h. No alcohol was offered in the intervening period (Saturday through Monday). This protocol was repeated weekly for 5 weeks. Tubes were weighed immediately before and after the exposure period. Volume consumed was converted to g/kg body weight of ethanol. Subsequent weekly 2 or 4 h measurements were averaged by strain and used as the dependent variable for data analysis and QTL mapping. The data were deposited in Gene Network (GN)^1^ and are available as traits 20010 through 20014 and 20077 through 20082 in the BXD Published Phenotypes database.

### Data Analysis

Statistical evaluation of daily alcohol intake was performed by ANOVA testing in R using the ***lm*** function for a 1 between-subjects variable (strain). Heritability at each time point was estimated from the ANOVA results by ss_strain_/ss_total_ (where ss = sum of squares, Belknap, 1998). Average weekly 2 h or 4 h intake was used to compute slopes and intercepts for each strain over the 5-week observation period.

Genetic correlational analyses among the DID phenotypes, between the DID phenotypes and BXD legacy phenotype data, and traditional quantitative trait loci (QTL) analysis were performed using a combination of R and GN software (Sloan et al., 2016)^1^. Traditional interval mapping in GN was performed using a simple regression method (Haley–Knott or HK) to compute QTL probability given strain genotypes and alcohol intake averaged by strain (Chesler et al., 2005; Mulligan et al., 2017). For traditional interval mapping, genome-wide suggestive (adjusted *p* < 0.63) and significant (adjusted *p* < 0.05) thresholds were determined based on 1,000 permutations of the trait data for each phenotype (GN default). The suggestive threshold is very permissive (see GN glossary of terms and features at www.genenetwork.org/glossary.html for more details) but strikes a balance between detection of false positives and highlighting loci that might be worth further investigation.

QTL analysis using genome-wide efficient mixed model association (GEMMA; Zhou and Stephens, 2012) was performed as a secondary method to traditional HK mapping in GN version 2 to assess the effect of population structure (kinship) between individuals. Full GEMMA Linear Mixed Model (LMM) support with the optional leave one chromosome out (LOCO) method was recently added to GN. GEMMA software is a computationally efficient LMM method for QTL mapping while explicitly accounting for genetic non-independence within each sample (Zhou and Stephens, 2012). Even for small sample sizes, after running Haley-Knott QTL mapping, GEMMA potentially allows for explorative fine-tuning of results at the SNP level. At each time point, and for each of 7,320 SNPs and phenotypes, we fitted the LMM using a kinship matrix K computed over the SNP genotypes. We also estimated significance thresholds with GEMMA using a permutation approach computing results 1,000 times while shuffling the phenotypes but keeping the genotypes and K the same (Churchill and Doerge, 1994). From every permutation we stored the highest Wald-test *p*-value in an ordered set and set the significance threshold at the 95th percentile and the suggestive threshold at the 67th percentile. For our data set, the average GEMMA significance threshold was LOD 4.1 and the average suggestive threshold was at LOD 3.3.

GEMMA was included as a mapping method for two main reasons. The first reason is that additional power can be gained when using mixed-model association methods. The increase in power results both from accounting for phenotypic covariance due to genetic similarity and by conditioning on associated markers as opposed to a single candidate locus (Yang et al., 2014). The second reason is that the impact of background structure on QTL detection has not been rigorously evaluated in BXD data sets. Thus, GEMMA can be applied in addition to traditional HK mapping to minimize detection of false positive QTLs resulting from kinship. QTLs identified by both approaches are unlikely to result from genetic similarity. Using both approaches has advantages over using a single mapping model because genetic relatedness is not addressed by the HK model and our data set is small (e.g., underpowered) for GEMMA.

For both HK and GEMMA QTL mapping methods, a 1.5 LOD drop from the top marker was used to define QTL confidence interval regions. Loci detected using traditional HK mapping (suggestive threshold or above) that were also detected using the secondary GEMMA method (LOD > 3) were considered for further analysis.

Enrichment analysis was performed using tools available at Enrichr (Kuleshov et al., 2016). Default settings were used for Enrichr.

B6 and D2 polymorphic genes with SNPs and/or small insertions/deletions (InDels) were identified using tools available at the Sanger Institute Mouse Genomes Project^2^ (Keane et al., 2011). The Sanger website provides annotations for variants and, for our analysis, a SNP or InDel was considered to be of potentially high impact if it was annotated as a “coding sequence variant,” “feature elongation,” “feature truncation,” “incomplete terminal codon variant,” “initiator codon variant,” “mature miRNA variant,” “missense variant,” “NMD transcript variant,” “regulatory region ablation,” “regulatory region amplification,” “regulatory region variant,” “splice acceptor variant,” “splice donor variant,” “splice region variant,” “stop gained,” “stop lost,” “TF binding site variant,” “TFBS ablation,” “TFBS amplification,” “transcript ablation,” or “transcript amplification.”

A large BXD database of hippocampal gene expression profiles generated from 67 naïve BXD strains [Hippocampus Consortium M430v2 (Jun 06); GN110; (Overall et al., 2009)] was used to prioritize candidate genes based on correlation with DID week 1 and 4 h phenotype data and cis expression QTL (eQTL) mapping.

For all candidate genes, literature associations between the term “alcohol” and each candidate gene were mined using the Chilibot^3^ website (Chen and Sharp, 2004).

## Results

### Initial Alcohol Consumption Is Variable and Heritable in the BXD Panel and Increases Over Time

**FIGURE 1:**
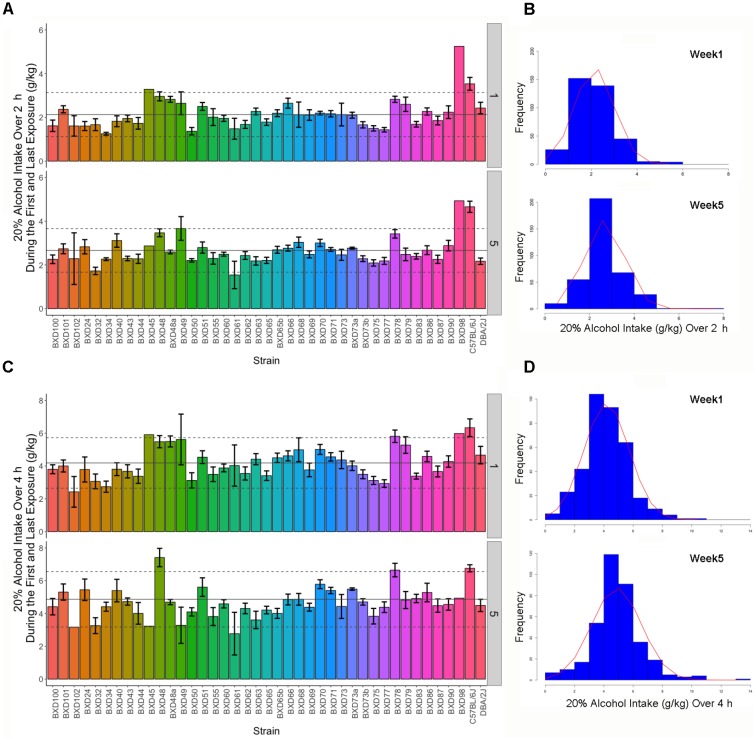
Strain distribution of 2 or 4 h alcohol intake over 5 weeks. **(A)** Alcohol intake of 20% (v/v) over a 2 h access period is shown in g/kg on the Y-axis by week and the X-axis lists the BXD strain numbers with B6 and D2 parental strains shown to the far right (bar graph, each color represents an inbred strain). Population mean and standard deviation are shown for each week by the solid and dashed horizontal line, respectively. Weekly 2 h intake was variable among BXD strains and increased over time. **(B)** Distribution (histogram) reflects week-averaged intake from individual B6, D2, and 39 BXD strains recorded over three consecutive days of 2 h exposure. The distribution of alcohol intake changed between weeks 1 and 5 indicating increased alcohol intake. **(C)** Alcohol intake of 20% (v/v) over a 4 h access period is shown in g/kg on the Y-axis by week and the X-axis lists the BXD strain numbers with B6 and D2 parental strains shown to the far right (bar graph). Alcohol intake of 20% alcohol during the 4 h access period (bar graph) was variable among BXD strains and increased over time. **(D)** Distribution (histogram) reflects intake from B6, D2, and 39 BXD strains recorded over a single 4 h exposure. The distribution of alcohol intake changed between weeks 1 and 5 indicating increased alcohol intake. For the 2 and 4 h exposure, alcohol intake varied by strain both within and across weeks. For both exposures, mean intake increased between weeks 1 and 5.

Intake of 20% alcohol for 2 or 4 h during the first and 5th week was variable in female B6, D2, and 39 BXD strains (**Figure 1**). Alcohol intake during the first 2 h exposure ranged from 1 to 3.55 g/kg compared to the last 2 h (week 5 day 3 or W5D3) exposure, which ranged from 1.67 to 5.01 g/kg. On the first 4 h exposure, alcohol intake ranged from 2.42 to 6.33 g/kg compared to intake on the last exposure, which ranged from 2.77 to 7.41 g/kg. ANOVA revealed a significant strain effect (all *p* < 0.0001) on intake for each time point with associated heritability estimates of 0.3 or greater, except for the first exposure on week 1 day 1 (W1D1) and W4D4 (**Table 1**). As expected, heritability on the first exposure to alcohol is lower than on subsequent exposure. Also as expected, heritability of 2 h weekly intake averaged for each strain over 3 days is much higher than strain-averaged 4 h intake resulting from a single measurement.

**Table 1:** Anova results table.

	2 h access						4 h access				
Strain Effect	W1D1	W1avg	W2avg	W3avg	W4avg	W5avg	W1D4	W2D4	W3D4	W4D4	W5D4
*R-*value	1.20E-09	<2.20E-16	<2.20E-16	<2.20E-16	<2.20E-16	<2.20E-16	1.22E-11	1.69E-13	2.89E-15	3.80E-08	2.06E-12
*F*-value	3.36	5.19	4.91	7.89	5.48	6.69	3.79	4.19	4.6	3.0	3.962
Df	39	40	40	40	40	40	40	40	40	40	40
h2	0.29	0.39	0.37	0.49	0.40	0.45	0.32	0.34	0.36	0.27	0.33

Average 2 or 4 h intake across all strains and the overall distribution of intake changed between week 1 and later weeks reflecting changes in drinking patterns that resulted from increased consumption over time (**Figure 1**). For 2 h intake, week was significantly associated (*p* < 0.0001; *R*^2^ = 0.039) with a 0.14 g/kg increase in alcohol intake per week (∼0.6 g/kg average increase in intake between weeks 1 and 5). For 4 h intake, week was significantly associated (*p* < 0.0001; *R*^2^ = 0.025) with a 0.2 g/kg increase in alcohol intake per week (∼0.8 g/kg average increase in intake between weeks 1 and 5). Pairwise correlations between initial daily, 2 h weekly average, and 4 h weekly alcohol intake tended to be higher between adjacent weeks and degrade as the interval between each week increases (**Supplementary Figure S1**).

### Genetic Differences in Alcohol Intake Over Time

Most strains demonstrated a gradual increase in alcohol intake over time (**Table 2**) during 2 or 4 h exposure. Strains with significantly increased 2 h intake over the 5-week period included BXD 40, 24, 49, 34, 50, 77, and 83. Of these strains, BXD 34, 50, 77, and 83 also demonstrated significantly elevated 4 h intake. Of note, some strains with high genetic similarity (∼80%, denoted by letters following strain name), such as BXD48 and BXD48a, showed highly divergent intake patterns over time with BXD48 showing significantly increased intake and BXD48a displaying stable or slightly decreased intake over time. The parental B6 strain demonstrated increased 2 h intake over the 5-week period and slightly decreased 4 h intake in contrast to the D2 strain, which showed relatively stable or slightly decreased intake for both the 2 and 4 h intake period. However, these changes in the parental strains were not statistically significant. Although modestly correlated (*r* = 0.41), change in 2 and 4 h intake over 5 weeks was not identical within strain. We observed a strong, negative correlation (-0.62) between slope (alcohol intake over time) and the y-intercept (baseline intake) for 4 h intake. As demonstrated in **Table 2**, strains with high initial intake tended to evince more stable or slightly decreased drinking over time. This negative relationship was present, albeit much weaker, for the 2 h time point (*r* = -0.25).

**Table 2:** Change in 2 and 4 h alcohol intake over 5-week.

	2 h			4 h		
Strain	Slope	Intercept	SE	Slope	Intercept	SE
BXD40	***0.329***	1.54	0.07	0.541	3.05	0.54
BXD24	***0.297***	1.51	0.07	0.545	3.20	0.55
C57BL/6J	0.278	3.20	0.11	-0.077	6.60	-0.08
BXD49	***0.246***	2.48	0.03	-0.455	6.01	-0.46
BXD34	***0.238***	1.16	0.04	***0.408***	2.46	0.41
BXD68	0.218	2.09	0.07	-0.002	4.96	0.00
BXD50	***0.211***	1.18	0.01	***0.232***	2.83	0.23
BXD77	***0.199***	1.22	0.02	***0.374***	2.47	0.37
BXD83	***0.197***	1.50	0.03	***0.408***	3.02	0.41
BXD62	***0.183***	1.57	0.03	0.153	3.59	0.15
BXD90	***0.169***	1.93	0.03	0.188	4.05	0.19
BXD73a	***0.168***	1.95	0.01	***0.362***	3.80	0.36
BXD75	***0.166***	1.21	0.04	0.198	2.62	0.20
BXD100	***0.161***	1.57	0.04	0.139	3.54	0.14
BXD70	0.160	2.23	0.06	0.132	4.88	0.13
BXD98	0.150	4.28	0.30	-0.318	6.87	-0.32
BXD73b	***0.146***	1.58	0.01	***0.319***	3.04	0.32
BXD78	***0.146***	2.61	0.03	***0.195***	5.72	0.19
BXD71	***0.144***	1.91	0.03	0.272	4.37	0.27
BXD60	***0.134***	1.75	0.02	***0.150***	3.81	0.15
BXD65b	***0.134***	1.96	0.04	-0.109	4.42	-0.11
BXD48	***0.134***	2.76	0.02	***0.542***	5.02	0.54
BXD44	0.127	1.74	0.06	0.296	2.91	0.30
BXD101	0.125	2.19	0.06	0.355	3.85	0.35
BXD87	0.120	1.58	0.04	***0.219***	3.23	0.22
BXD73	0.118	1.94	0.04	0.060	4.28	0.06
BXD65	***0.116***	1.73	0.03	0.232	3.36	0.23
BXD61	0.109	1.21	0.08	-0.372	3.90	-0.37
BXD43	***0.107***	1.83	0.03	***0.288***	3.39	0.29
BXD102	0.103	1.14	0.19	0.183	1.84	0.18
BXD86	***0.103***	2.09	0.03	0.218	4.13	0.22
BXD69	***0.101***	2.03	0.03	0.197	3.58	0.20
BXD55	0.088	1.79	0.04	0.122	3.17	0.12
BXD51	0.080	2.36	0.03	0.379	4.08	0.38
BXD66	0.047	2.47	0.03	0.085	4.33	0.09
BXD32	0.018	1.61	0.01	0.075	2.73	0.07
BXD4S	0.007	2.56	0.21	-0.356	5.57	-0.36
BXD79	-0.017	2.61	0.03	-0.080	5.25	-0.08
BXD63	-0.026	2.25	0.05	-0.125	4.52	-0.13
DBA/2J	-0.041	2.17	0.09	-0.040	4.32	-0.04
BXD48a	-0.046	2.89	0.05	-0.264	5.72	-0.26

*Slope (g/kg per week) and associated y-intercept (g/kg) and standard error (SE) are shown for the 2 or 4 h exposure period by strain. Slopes sorted by 2 h exposure period. All slopes calculated from strain-averaged intake (4 h g/kg) or average weekly intake by strain (2 h g/kg). Bold italic slope values indicate significant (p < 0.05) effect of week on g/kg alcohol intake after linear regression. Intake is variable but generally increases over time for both the 2 and 4 h exposures in the BXD strains. However, slope at 2 h is not always identical to slope at 4 h and both traits are modestly correlated (r = 0.41). Parental strains are highlighted*.

### Identification of QTLs for Alcohol Intake

We first assessed whether there were QTLs associated with the heritable variation in initial (week 1) or sustained weekly alcohol intake using two different mapping methods (traditional HK and GEMMA to correct for kinship). As expected, based on the inclusion of 39 strains, no significant QTLs were detected following multiple test correction (**Supplementary Table S2**). Using a suggestive threshold, seven QTLs were identified for HK and GEMMA (**Table 3**). QTLs detected by both methods are expected to be more robust than QTLs detected by HK alone, because these QTLs do not arise as a result of genetic relatedness. We prioritized the overlapping QTLs detected by both methods for further analysis. In all seven cases, inheritance of the B6 parental allele was associated with higher 2 or 4 h alcohol intake. QTLs on Chrs 2, 6, and 12 were only identified for 4 h intake on week 1. In contrast, overlapping QTLs on Chr 4 were only identified for 2 h intake on weeks 2 and 3, and the QTL on Chr 8 was identified for 4 h intake on week 3 and 2 h intake on week 5.

**Table 3:** Suggestive QTL detected by multiple mapping methods.

Time	Method	Chr	Locus	Mb	Additive Effect	LOD	CIMb (left)	CIMb (right)
*W1.4 h*	*HK*	2	*rs13476358*	*16.306*	*–0.459*	*2.43*	*9.336*	*42.674*
*W1.4 h*	*GEMMA*	2	*Affy_PC2_15*	*15.000*	***–***	*3.07*	*9.000*	*30.441*
*W2.2 h*	*HK*	*4*	*rs32939068*	*153.934*	*–0.247*	*2.46*	*151.094*	*156.101*
*W2.2 h*	*GEMMA*	*4*	*rs32939068*	*153.934*	***–***	*3.44*	*153.372*	*155.226*
*W3.2 h*	*HK*	*4*	*rs32939068*	*153.934*	*–0.303*	*2.81*	*151.094*	*156.101*
*W3.2 h*	*GEMMA*	*4*	*rs32939068*	*153.934*	***–***	*4.08*	*153.372*	*155.493*
*W1.4 h*	*HK*	*6*	*rs30422489*	*91.707*	*–0.508*	*3.08*	*89.011*	*93.600*
*W1.4 h*	*GEMMA*	*6*	*rs30422489*	*91.707*	***–***	*3.23*	*89.011*	*93.600*
*W3.4 h*	*HK*	*8*	*rs49907965*	*89.094*	*–0.573*	*2.67*	*80.460*	*95.747*
*W3.4 h*	*GEMMA*	*8*	*rs49907965*	*89.094*	***–***	*3.16*	*86.380*	*95.747*
*W5.2 h*	*HK*	*8*	*rs49907965*	*89.094*	*–0.325*	*2.77*	*82.870*	*95.736*
*W5.2 h*	*GEMMA*	*8*	*rs49907965*	*89.094*	***–***	*3.23*	*82.870*	*95.747*
*W1.4 h*	*HK*	*12*	*rs3717933*	*11.076*	*–0.488*	*2.76*	*9.864*	*15.820*
*W1.4 h*	*GEMMA*	*12*	*rs49972008*	*12.603*	***–***	*3.07*	*7.991*	*26.035*
*Slope. 2 h*	*HK*	*15*	*rs31691968*	*63.392*	*0.045*	*3.19*	*58.000*	*67.990*
*Slope. 2 h*	*GEMMA*	*15*	*rs31691968*	*63.392*	***–***	*3.69*	*58.000*	*67.990*

*QTLs were mapped for each week (W) of 2 or 4 h alcohol intake or for progressive 2 or 4 h alcohol intake based on slope analysis over five weeks of the DID paradigm. Additive effect, based on HK, mapping, is the contribution of parental alleles to mean trait expression. Negative additive values are associated with higher expression of the B6 allele. CI indicates the 1.5 LOD drop confidence interval. Text color indicates whether higher intake is driven by the inheritance of the B6 (blue) or D2 (red) parental allele at the locus. All QTL were detected at the suggestive threshold.*

Next, we identified QTLs for progressive alcohol intake using slope analysis of 2 or 4 h intake over 5-weeks. No significant or suggestive QTLs were identified for progressive 4 h intake. However, a suggestive QTL on Chr 15 was identified for progressive 2 h alcohol intake using both HK and GEMMA mapping methods (**Table 3**). In contrast to the other seven QTLs, the 2 h progressive intake QTL was associated with higher intake and inheritance of the D2 parental allele.

### Identification of Candidate Genes Driving Initial Alcohol Intake

The majority of suggestive overlap QTLs detected were associated with variation in initial 4 h alcohol intake on week 1 (W1.4 h, **Table 3**). Thus we prioritized these QTLs on Chrs 2 (9–30.4 Mb), 6 (89–93.6 Mb), and 12 (9.8–15.8 Mb) for identification of candidate genes driving variation in initial early alcohol intake. Hereafter, all QTLs are referred to by their QTL (Q) Chr number. The number of candidate genes located within each confidence interval varies [Q2 = 325, Q6 = 54, Q12 = 25; based on mouse assembly GRCm38/mm10 and UCSC RefSeq (refGene) Table Browser annotations, **Supplementary Table S3**]. Using genome sequence data generated for the parental strains we identified all genes (excluding gene models, predicted genes, non-coding and pseudo-genes) within each interval that were polymorphic and overlapped by higher impact sequence variants (see Methods). This reduced the number of candidate genes within each interval to Q2 = 60, Q6 = 13, and Q12 = 4 (**Supplemental Table S4**). These genes were further prioritized using naïve hippocampal expression data generated for the BXD family (**Table 4** and **Supplemental Table S5**). For Q2, *Arhgap21, Gpr158, Mrpl41, Myo3a, Entpd4, Lhx3, Rapgef1*, and *Nup188* were all significantly correlated with alcohol intake during the first 4 h exposure in week 1, and the expression of each candidate was also regulated by local sequence variants (cis eQTL). Top candidates for Q6 included *Adamts9* and *Fgd5*. For Q12, *Nt5c1b* was the only candidate that contained putative high impact variants and whose expression covaried with 4 h alcohol intake on week 1. Products of all candidate genes in Q2, Q6, and Q12 played a role in signal transduction, metabolism, development, or endothelial cell response.

**Table 4:** Prioritized QTL candidate genes.

Symbol	Description	Gene Wiki	QTL	HIP:Mean Expr	HIP:cis eQTL	HIP:Trait Correlation
Dnajc1	DnaJ (Hsp40) homolog. subfamily C, member 1	GRP78 partner in ER; translation, beneficial effects in ER stress induced metabolic dysfunction	Q2: Initial	8.74	Sug	–
*Armc3*	armadillo repeat containing 3	?	Q2: Initial	**5.64**	Sig	–
***Arhgap21***	Rho GTPase activating protein 21	Signaling	Q2: Initial	11.76	Sig	Sig
***Gpr158***	G protein-coupled receptor 158	Regulator of RGS complexes and Rgs7 signaling in brain; human variants may influence energy expenditue and metabolism	Q2: Initial	6.51	Sig	Sig
*Myo3a*	Myosin IIIA	Acin based motor protein with kinase activity	Q2: Initial	6.35	–	Sig
***Mrpl41***	Mitochondrial ribosomal protein L41	Regulation of cell death	Q2: Initial	10.29	Sig	Sig
*Uap1l1*	UDP-N-acetylglucosamine pyrophorylase 1 like 1	?	Q2: Initial	9.01	Sig	–
***Entpd2***	Ectonucleoside triphosphate diphosphohydrolase 2	Metabolism	Q2: Initial	9.72	Sig	Sig
*Ptgds*	Prostaglandin D2 synthase	Glucose and insulin metabolism; regulated by estradiol; inflammatory response	Q2: Initial	13.90	Sig	–
*C8g*	Complement component 8, gamma subunit	Immune function	Q2: Initial	7.13	Sig	–
*Kcnt1*	Potassium channel, subfamily T, member 1 (slack, low threshold slowly adapting)	Localized to the postsynaptic density; involved in learning and memory and initial response to novel situations and environments	Q2: Initial	8.98	Sig	–
*Lhx3*	LIM homeobox protein 3	Trancription factor activity, nervous system development	Q2: Initial	6.23	–	Sig
*Egfl7*	EGF-like domain 7	Development	Q2: Initial	7.81	Sug	–
*Rapgef1*	Rap guanine nucleotide exchange factor (GEF) 1	Signaling; GABAergic neuronal development	Q2: Initial	7.41	Sug	Sig
*Nup188*	Nucleoporin 188	Development	Q2: Initial	8.50	–	Sig
*Plxna1*	plexin A1	Development; signaling	Q6: Initial	9.77	Sig	–
*Fgd5*	FYVE, RhoGEF and PH domain containing 5	Endothelial specific gene	Q6: Initial	8.00	–	Sig
*Adamts9*	A disintegrin-like and metalloprotease (reprolysin type} with thrombospondin type 1 motif, 9	Endothelial and vascular response	Q6: Initial	9.38	Sug	Siq
*Nt5c1b*	5′-nucleotidase, cytosolic 1B	Metabolism	Q12: Initial	7.18	Sug	Sig
***Per3***	Period 3	Circadian signaling	Q4: Sustained	7.11	Sig	Sig
***Prdm16***	PR domain containing 16	Metabolism; brown versus white fat differentiation	Q4: Sustained	6.52	Sig	Sig
*Tnfrsf14*	Tumor necrosis factor receptor superfamily, member 14 (herpesvirus entry mediator)	Inflammation and immune response	Q4: Sustained	**5.20**	Sig	–
***Cyld***	Cylindromatosis (turban tumor syndrome}	Immune; regulator of NF-kappaB signaling	Q8: Sustained	10.41	Sig	Sig
*Chd9*	Chromodomain helicase DNA binding protein 9	Expressed in osteoprogenitors	Q8: Sustained	9.55	Sig	–
***Aktip***	AKT interacting protein	Ft1 protein; telomere maintenance; development	Q8: Sustained	7.49	Sig	Sig
***Fto***	Fat mass and obesity associated (alpha-ketoglutarate-dependent dioxygenase FTO)	Metabolism; energy homeostasis; regulator of adipogenesis	Q8: Sustained	6.94	Sig	Sig
***Crnde***	Colorectal neoplasia differentially expressed (non-protein coding}	Metabolism; control of glucose and lipid metabolism	Q8: Sustained	6.63	Sig	Sig
***Mmp2***	Matrix metaloproteinase 2	Immune system; inflammatory response	Q8: Sustained	6.66	Sig	Sig
***Coq9***	Coenzyme QB	Metabolism: deficiency impacts mitochondrial function	Q8: Sustained	10.49	Sig	Sig
***Kifc3***	Kinesin family member C3	Peroxisomal transport	Q8: Sustained	10.01	Sig	Sig

*High priority candidate genes selected based on regulation of expression by local sequence variants (cis eQTL) and co-expression with DID intake traits (Slope2 hWeek, 2 h.W3avg, 2 h.W5avg, and W1.4 h). See **Supplementary Table S5** for full results. Gene expression signatures for each gene generated on the Affymetrix M430 platform for BXD hippocampus (HIP, naïve to treatment). Genes in bold have hippocampal gene expression that is significantly regulated by a cis eQTL (Sig) and significantly (p < 0.05) coexpressed (Sig; based on Pearson’s r-value) with DID alcohol intake traits. Bold text in the HIP:MeanExpr column used to indicate genes with very low expression in hippocampus. In the hippocampal data set mean expression is 8 ± 2 log_2_ units. Function summarized using GN Gene Wiki tool.*

### Identification of Candidate Genes Driving Variation in Sustained Weekly Alcohol Intake

The same strategy used to identify candidates driving variation in initial consumption was used to identify candidates on Q4 (151–155 Mb) and Q8 (86.4–95.7 Mb) associated with variation in 2 h intake on weeks 2 and 3, and variation in 4 h intake on week 3 and 2 h intake on week 5, respectively. The number of candidates within each interval was 51 for Q4 and 93 for Q8 (**Supplementary Table S3**). The number of candidate genes overlapping higher impact variants within each locus was 36 for Q8 and 16 for Q4 (**Supplementary Table S4**). For the Q4 interval, the highest priority candidates based on genetic cis-regulation and co-variation analysis using hippocampal expression data were *Per3* and *Prdm16* (**Table 4** and **Supplementary Table S5**). Top candidates for the Q8 interval included *Cyld, Aktip, Fto, Crnde, Mmp2, and Kifc3*. All of these high priority candidate genes were involved in circadian signaling, metabolism, or immune response.

### Identification of Candidate Genes and Pathways Driving Variation in Progressive Alcohol Intake

Variation in progressive 2 h alcohol intake was associated with a suggestive QTL on Q15 (58–68 Mb) that contained 54 candidate genes (**Supplementary Table S3**). Only four of these genes (*Adcy8, Tg, Ndrg1*, and *Wisp*) were overlapped by variants of predicted higher impact (**Supplementary Table S4**). None of these candidates were modulated by cis eQTLS in naïve BXD hippocampus or correlated with the slope for 2 h alcohol intake over 5 weeks (**Supplemental Table S5**).

### Trait Covariation With Initial Alcohol Intake

**FIGURE 2:**
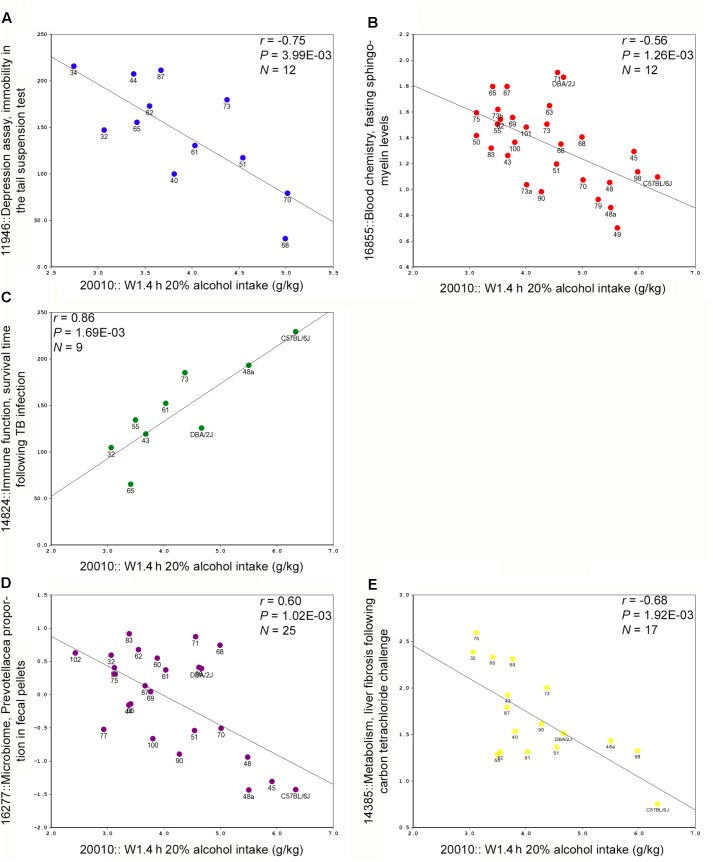
Representative scatterplots representing correlations between BXD legacy trait data and alcohol intake during the first 4 h exposure on week 1. **(A)** A measure of depression (immobility in the tail suspension test) is negatively correlated with alcohol intake. **(B)** Fasting sphingomyelin levels are also negatively correlated with intake. **(C)** A measure of immune function, infection survival time is positively correlated with intake. **(D)** Measures of one member of the gut microbime are negatively correlated with intake. **(E)** A metabolic/immune trait, liver injury following insult, is negatively correlated with intake.

Trait data have been collected for the BXDs since the generation of the first BXD cohort in the late 1970’s and much of this data is available in the BXD Published Phenotypes database available on GN. We queried this database to retrieve traits that were significantly correlated with W1.4 h, a heritable and QTL modulated trait associated with alcohol intake at early exposure. Forty-one traits were significantly correlated (*p* < 0.005) with the first 4 h alcohol intake trait. Representative scatterplots are shown in **Figure 2**. Correlated traits were subdivided into six categories: behavior; blood and brain chemistry, and hematology; drug response (morphine and alcohol); immune function; metabolism, and microbiome (**Supplementary Table S6**).

## Discussion

Here, we report large variability in 2 and 4 h alcohol consumption in B6, D2, and 39 BXD strains using a DID protocol over a 5-week time period. BXD strains demonstrated high, low, or intermediate alcohol intake relative to parental strains (**Figure 1**). Regression analysis revealed that, in general, alcohol intake increased from weeks 1 to 5 regardless of session duration. Although it is important to note that some strains demonstrated greatly increased intake while others remained relatively stable or decreased slightly over the 5-week period. Our study both replicated previous findings on DID alcohol intake and generated novel insight into progressive drinking. In contrast to our study, most of the previous DID studies in male and female inbred strains focused primarily on the first few weeks of DID. These studies reported higher intake in B6 compared to D2, and that alcohol consumption “stabilized” by the second day of access (Rhodes et al., 2005). When these studies were extended for 12 consecutive days in male B6 mice, intake levels were reported to be constant (Rhodes et al., 2005). We also reported stabilization of alcohol intake early in week 1 in that heritability was reduced at first exposure compared to subsequent exposures. This was a result of greater within strain variation upon first experience with alcohol. Accordingly, we also observed much higher intake of 20% alcohol at 2 and 4 h in the B6 parental strain relative to D2. By lengthening the DID protocol to 5 weeks, our work extends the findings of previous studies and we reported increased consumption and, at least for 2 h intake, higher heritability (less within strain variation) during later weeks (3, 4, and 5). Similar increases in intake have been reported for B6 males over a 14-day DID access period (Linsenbardt and Boehm, 2014). Taken together, these results provide evidence that extended access DID protocols of 2 weeks or longer result in increased alcohol intake in B6J. For the first time, we report that this trend of enhanced consumption during extended DID is also apparent in the BXD family of strains. Patterns of initial weekly and progressive intake during 2 or 4 h sessions over 5 weeks varied between B6, D2, and the BXD strains. This variability was heritable and we were able to identify several suggestive loci for variation in initial intake during week 1, and variation in sustained and progressive intake.

The DID paradigm typically employs both a 2 h and a longer 4 h access period. In our study, some strains displayed marked differences in alcohol intake between access periods and identified QTLs were not always consistently detected for both periods. There are several explanations for the lack of complete congruency between the 2 and 4 h measures. First, the 2 h time point is a repeated measure and is thus a much more stable measure of weekly progressive intake. In addition, the extended 4 h access time period probably reflects a larger behavioral repertoire related to differences in learning and consummatory behavior. For example, male B6 mice allowed to drink alcohol in a modified DID paradigm in which they were given a 2 h access period over 14 consecutive days not only increased their intake over sessions, but also learned to “front load” their intake, and consumed the majority of their alcohol on the last session within the first few minutes of the 2 h access period (Linsenbardt and Boehm, 2014). In our study using female mice, we observed increased intake over the 5-week period in B6 females for the 2 h time point (**Table 2**), but the same trend was not observed for the 4 h time point. However, several strains, such as BXD 40, 29, and 34, showed increased intake over 5 weeks for both the 2 and 4 h sessions. It is unknown whether B6 females display front loading, but differences in this behavior, or a ceiling effect on intake in the 4 h access period, may be an additional source of variation that contributes to differences between the 2 and 4 h access period between and within strain in our study. For genetic mapping, the 2 h trait has higher heritability and is more amenable to mapping. However, it is also possible that different genetic factors control each trait. Temporal dissection of intake over the 2 and 4 h access period for strains with similar and dissimilar 2 and 4 h intake would be needed to reconcile some of these differences.

Our study included 39 BXDs strains with a variable number of replicates within strain (1–16). Traditional QTL mapping incorporates the mean of the dependent measure for each BXD strain. Power to detect QTLs is derived largely from the number of strains (Belknap, 1998; Andreux et al., 2012). However, the traditional mapping method used for BXD data does not account for population structure and may result in false positives. To account for this, we mapped QTLs using both a simple regression method (HK) in GN that does not account for kinship, and another mapping algorithm (GEMMA) available in GN, version 2, that does. Even though GEMMA is not typically used for smaller data sets, several QTLs were replicated using both methods, albeit at a suggestive level. Both methods used the same marker panel and dependent variables for alcohol intake. QTLs detected by both methods are expected to be more robust against identification of false positives due to genetic relatedness compared to using either model alone (see methods). With 39 strains and a variable number of replicates within strain, our study was only powered to detect QTLs of large effect. As expected, we did not identify any significant loci. However, using both mapping methods we identified: suggestive QTLs associated with variation in alcohol intake during early exposure (W1.4 h) on Chr 2, 6, and 12, and suggestive QTLs associated with variation in sustained intake during later weeks on Chrs 4 and 8. We were also able to identify a QTL on Chr 15 associated with progressive alcohol intake. To assess whether these QTLs are likely to reach significance if more strains are added we winsorized (transformation of the data by limiting extreme values) the existing 39 strain data to better fit assumptions of normality assumed when using HK and GEMMA mapping methods. Winsorizing the data resulted in higher LOD scores for all QTLs for both mapping methods. This suggests that, assuming a normal trait distribution in the BXD population, testing a larger number of BXD strains will result in significant LOD scores. Thus, the provisional QTLs identified in our study are worth investigating further and may contain genes that drive higher or lower initial or sustained alcohol consumption. As such, we used multiple lines of evidence based on overlapping gene variants and gene expression to nominate candidate genes for suggestive QTLs identified in this study. An important caveat of this approach is that mutations that impact protein function without modulating gene expression will be missed. However, this is a powerful and integrative approach that nominates the best candidates based on all available data. Candidates associated with genetic predisposition for high or low intake during the 1st week of exposure to alcohol included *Arhgap21, Gpr158, Mrpl41, Myo3a, Entpd2, Lhx3, Rapgef1*, and *Nup188* (Q2); *Adamts9* and *Fgd5* (Q6); and *Nt5c1b* (Q12). All candidates were overlapped by variants that may impact gene regulation or function, and their expression in hippocampus was cis-modulated and correlated with 4 h intake during week 1. Therefore, pre-existing patterns of expression in these genes due to the presence of functional gene variants could alter initial alcohol intake behavior. Only one of these genes, Entpd2, had been directly implicated in alcohol-related responses in animal model systems (Rico et al., 2008). Rico and colleagues found that acute alcohol exposure led to alterations in brain nucleotide (specifically, ATP hydrolysis) metabolism *in vivo* and a reduction in the mRNA level of the zebrafish homolog of ENTPD2 (NTPDase2). Although not directly associated with alcohol related traits in human or animal model systems, *Nt5c1b* was also involved in nucleotide metabolism (specifically, adenosine formation following ATP hydrolysis; Sala-Newby and Newby, 2001). Other candidates were involved in intracellular signaling pathways (*Arhgap21, Gpr158, Rapgef1, Adamts9, Fgd5, Mrpl41, Nt5c1b*) that mediated diverse biological processes related to metabolism, G-protein coupled receptor signaling, and endothelial response (**Table 4**). Taken together, these results suggest that alterations in ATP metabolism and other signaling pathways could drive variation in initial alcohol intake in the DID paradigm.

Using a similar approach we also identified *Per3* and *Prdm16* (Q4) and *Cyld, Aktip, Fto, Crnde, Mmp2, and Kifc3* (Q8) as high priority candidate genes for variation (higher or lower) in sustained intake during later weeks. Variation in 2 h intake on weeks 2 and 3 was associated with Q4, and variation in 4 h intake on week 3 and 2 h intake on week 5 was associated with Q8. Importantly, the Q4 and Q8 intervals were detected at multiple time points over the course of the 5-week DID study. These concordant and reproducible QTLs are less likely to represent false positives. Candidates for the Q4 and Q8 interval play key roles in circadian signaling (*Per3*), metabolic (*Prdm16, Fto, Crnde*), and immune response (*Mmp2, Cyld*). Alterations in metabolism (e.g., mitochondrial dysfunction, insulin/glucose dysregulation), circadian cycle, sleep disruptions, and inflammation have long been associated with chronic alcohol use, abuse, and alcoholism. In particular, several of these genes, *Per3* (Q4) and *Fto*, and *Mmp2*(Q8) have all been previously associated with ethanol response. In human populations, PER3 variants were associated with insomnia severity in alcohol-dependent patients (Brower et al., 2012). In the BXD panel, a promoter mutation in *Per3* was associated with expression variation in naïve animals (cis-modulation) as well as variation in alcohol-related traits and expression changes following stress and alcohol exposure (Wang et al., 2012). Altered circadian expression of *Per3* was also associated with administration of a liquid alcohol diet in rats (Chen et al., 2004). Another strong candidate for sustained intake was *Fto*, a gene regulating fat mass, located on Chr 8 and associated with variation in intake during week 5. *Fto* variants were associated with frequency of alcohol consumption (Sobczyk-Kopciol et al., 2011; Young et al., 2016) and alcohol dependence (Wang et al., 2013) in human populations. *Mmp2*, also located on Chr 8, is known to be regulated by alcohol exposure in numerous human and animal systems and in many different cell and tissue types (Partridge et al., 1999; Burnham et al., 2007; Fiotti et al., 2008; Peng et al., 2013), however, a role for this gene in regulating alcohol intake is unclear. Here we report *Per3* and *Fto* as a strong candidate genes located on Chr 4 and Chr 8, respectively, that may underly variation in sustained alcohol intake during DID in the BXD family. Both were associated with alcohol-related traits in human and rodent model systems, and their expression was correlated with 2 h intake during week 3 (negative correlation, *Per3*) or week 5 (positive correlation, *Fto*) of DID. In addition, *Per3* contains functional polymorphic regulatory variants known to impact expression in the BXD panel and *Fto* is a cis-modulated gene containing multiple missense variants between B6 and D2.

By extending the DID paradigm to a longer 5-week period we were also able to identify a suggestive QTL on Chr 15 for variation (higher or lower) in progressive alcohol intake. None of the genes located within the QTL confidence interval was cis-modulated in BXD hippocampus. This was an expected negative result based on the use of an expression data set generated from naïve animals. However, there were only a handful of genes (*Adcy8, Tg, Ndgr1*, and *Wisp*) within this interval that contained variants likely to impact gene regulation or function. Of these, only *Adcy8* was previously associated with alcohol-related traits (Procopio et al., 2013). Procopio and colleagues reported that a haplotype within the coding region of ADCY8 was associated with alcohol dependence co-occurring with depression in females. Given that progressive alcohol intake likely reflects changing alcohol consumption over time, it is reasonable to expect that the underlying gene variants may interact with alcohol exposure to produce variation in intake over time. Of interest, genes located within the Q15 interval were significantly enriched (adjusted *p* < 0.05) for HDAC2 and PPARD binding based on consensus genome-wide ChIP-X data from ENCODE and integrated ChIP Enrichment Analysis (ChEA). None of the other QTL intervals (Q2, Q4, Q6, Q8, or Q12) were enriched for transcription factor binding or histone modifications based on Enrichr analysis, suggesting that gene variants in the Q15 region may exert their effect through alcohol experience dependent epigenetic modulation.

This is the first work of its kind to elucidate genetic influence on ethanol consumption at first opportunity and repeated opportunities over several weeks in a large genetic reference population of mice. As in other studies, the DID paradigm has been applied successfully to reveal genetic influences on alcohol consumption and the impact of alcohol intake on brain gene transcription (Rhodes et al., 2005, 2007; Mulligan et al., 2011; Thiele and Navarro, 2014; Thiele et al., 2014). The DID paradigm allows the animal to achieve significant blood ethanol concentrations, i.e., binge drinking. Indeed, we found that some strains consume large amounts of alcohol and that most of the strains studied increased their consumption by more than 0.5 g/kg. However, a caveat of our study is that we did not measure blood alcohol concentrations (BACs) weekly. We did not attempt to obtain BACs because of the added stressor of blood collection. However, this precludes an analysis of metabolic effects that may have contributed to changes in alcohol intake among strains.

One of the great advantages of a systems approach in the BXD panel is the ability to find associations between a trait of interest and other phenotypes generated in-house, and by others, in the extensive BXD trait database available at GN. Not only were we able to nominate candidate genes for suggestive QTL related to initial, sustained, and progressive alcohol intake, but we were also able to leverage 1000s of legacy trait records available for the BXD strains to associate variation in initial alcohol consumption with variation in metabolic, metagenomic, immune, and behavioral traits in the BXDs (**Supplementary Table S4**). Some of these associations were expected (e.g., correlations with depression- and anxiety-related traits and alcohol intake; and covariation with morphine and other alcohol response traits), while others were novel and may uncover new areas of interest. For example, and perhaps most surprising, a negative correlation between components of the gut microbiome and initial 4 h alcohol intake (**Figure 2D**). Of interest, Prevotellaceae was found to be enriched in fecal samples from alcohol cirrhosis patients relative to control samples (Chen et al., 2011). Oral levels of Lactobacillales and Prevotellaceae were also altered following alcohol intake in humans (Fan et al., 2018). Taken together these novel findings could indicate the presence of latent relationships between alcohol-related diseases and members of the microbial community. This opens the research area to what these associations mean in the general physiology and behavior of mice and other species, by hypothesis generation. In conclusion, we have shown here that there is a large genetic component to both initial, sustained, and progressive consumption of alcohol in the DID paradigm among the BXD family of mice. Moreover, through forward genetic mapping and systems biology, we have identified candidate genes and pathways likely to mediate high alcohol intake.

## Author Contributions

MKM, MD, and BCJ wrote the manuscript. MKM, DA, PP, WZ, and BCJ analyzed the data. LL, BCJ, WZ, MKM, ET, SAC, and PM designed and implemented the study.

## Conflict of Interest Statement

The authors declare that the research was conducted in the absence of any commercial or financial relationships that could be construed as a potential conflict of interest.

## Footnotes

1 www.GeneNetwork.org

2 http://www.sanger.ac.uk/sanger/Mouse_SnpViewer/

3 www.chilibot.net

## Supplementary Material

FIGURE S1 Bivariate correlations of alcohol intake at initial exposure and across 2 or 4 h weekly intake. Correlations based on weekly (W) and daily (D) intake (g/kg) for individuals from B6, D2, and 39 BXD strains. Data averaged by strain for the first day of 2 h alcohol exposure (2 h:W1D1) and for each 4 h exposure. Data averaged by strain and week for subsequent 2 h exposures. All correlations were positive. Intake at first exposure for 2 h on W1D1 is highly correlated with subsequent average 2 and 4 h weekly intake. Correlations are higher between adjacent weeks and generally decay slightly over time. Compared to correlations between 4 h intake on the first and last exposure, correlations between intake at first (W1D1 or W1avg) and last exposure are higher for 2 h intake.

TABLE S1 Strain and replicate summary.

TABLE S2 QTL comparison table.

TABLE S3 Candidate genes located within QTL confidence intervals.

TABLE S4 Candidate genes within QTL confidence intervals overlapped by higher impact sequence variants.

TABLE S5 Full list of high priority candidate genes and evidence for selection.

TABLE S6 BXD trait correlations.
