# Supplementary figures and images for: Genetic Contribution to Initial and Progressive Alcohol Intake Among Recombinant Inbred Strains of Mice
Source: Front Genet. 2018 Sep 25;9:370. doi: 10.3389/fgene.2018.00370 (PMC6167410; doi:10.3389/fgene.2018.00370)

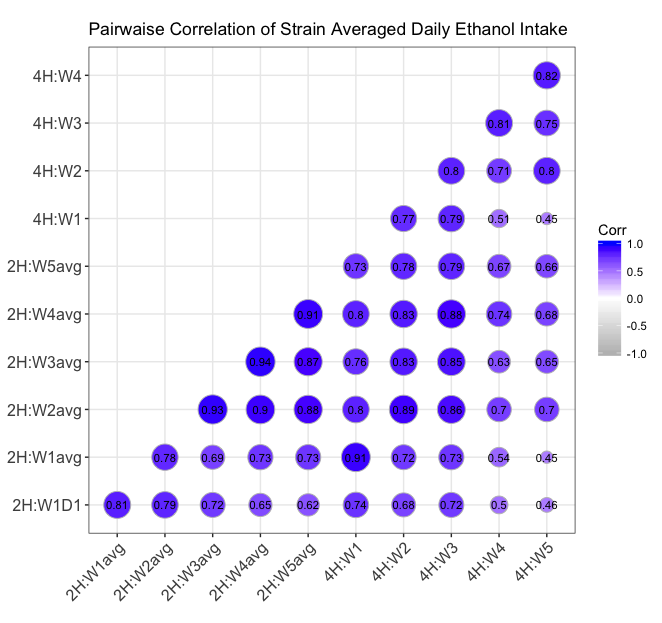

Supplement: FIGURE S1 — Bivariate correlations of alcohol intake at initial exposure and across 2 or 4 h weekly intake. Correlations based on weekly (W) and daily (D) intake (g/kg) for individuals from B6, D2, and 39 BXD strains. Data averaged by strain for the first day of 2 h alcohol exposure (2 h:W1D1) and for each 4 h exposure. Data averaged by strain and week for subsequent 2 h exposures. All correlations were positive. Intake at first exposure for 2 h on W1D1 is highly correlated with subsequent average 2 and 4 h weekly intake. Correlations are higher between adjacent weeks and generally decay slightly over time. Compared to correlations between 4 h intake on the first and last exposure, correlations between intake at first (W1D1 or W1avg) and last exposure are higher for 2 h intake. [file Image_1.TIF]
